# TwinF interface inhibitor FP802 prevents retinal ganglion cell loss in a mouse model of amyotrophic lateral sclerosis

**DOI:** 10.1186/s40478-024-01858-0

**Published:** 2024-09-12

**Authors:** Yu Meng Wang, Jing Yan, Sarah K. Williams, Richard Fairless, Hilmar Bading

**Affiliations:** 1 Department of Neurobiology, Interdisciplinary Center for Neurosciences (IZN), Heidelberg University, 69120 Heidelberg, Germany; 2 Department of Neurology, University Clinic Heidelberg, 69120 Heidelberg, Germany; 3 Clinical Cooperation Unit (CCU) Neurooncology, German Cancer Consortium (DKTK), German Cancer Research Center (DFKZ), 69120 Heidelberg, Germany; 4 Present Address: FundaMental Pharma GmbH, 69120 Heidelberg, Germany

**Keywords:** Amyotrophic lateral sclerosis, Retinal ganglion cell degeneration, Neuroprotection, TwinF interface inhibitor, FP802, Neuroprotective gene regulation

## Abstract

Motor neuron loss is well recognized in amyotrophic lateral sclerosis (ALS), but research on retinal ganglion cells (RGCs) is limited. Ocular symptoms are generally not considered classic ALS symptoms, although RGCs and spinal motor neurons share certain cell pathologies, including hallmark signs of glutamate neurotoxicity, which may be triggered by activation of extrasynaptic NMDA receptors (NMDARs). To explore potential novel strategies to prevent ALS-associated death of RGCs, we utilized inhibition of the TwinF interface, a new pharmacological principle that detoxifies extrasynaptic NMDARs by disrupting the NMDAR/TRPM4 death signaling complex. Using the ALS mouse model SOD1^G93A^, we found that the small molecule TwinF interface inhibitor FP802 prevents the loss of RGCs, improves pattern electroretinogram (pERG) performance, increases the retinal expression of *Bdnf*, and restores the retinal expression of the immediate early genes, *Inhibin beta A* and *Npas4*. Thus, FP802 not only prevents, as recently described, death of spinal motor neurons in SOD1^G93A^ mice, but it also mitigates ALS-associated retinal damage. TwinF interface inhibitors have great potential for alleviating neuro-ophthalmologic symptoms in ALS patients and offer a promising new avenue for therapeutic intervention.

## Introduction

Amyotrophic lateral sclerosis (ALS), a devastating and non-treatable neurodegenerative disorder, is characterized by the progressive loss of motor neurons, leading to muscle weakness and paralysis [5]. While traditionally associated with spinal cord and cortical motor neuron degeneration, ALS also affects the eyes, with several studies revealing significant thinning of the retinal layers containing both retinal ganglion cell (RGC) bodies and axons [7, 25]. This phenomenon has been observed not only in ALS patients but also in the widely used SOD1^G93A^ ALS mouse model, where significant RGC loss is also reported [24]. SOD1^G93A^ mice have been instrumental in understanding ALS disease mechanisms, since they closely mimic human disease progression and have been extensively used in the search for therapies for ALS [29]. However, the development of effective treatments for ALS remains a significant challenge, with very few approved disease-modifying therapies available, providing only modest survival benefits of a few months [32]. FP802, a so-called TwinF interface inhibitor, has recently emerged as a promising drug candidate, offering neuroprotection of spinal motor neurons in SOD1^G93A^ mice, resulting in a reduction in the serum biomarker neurofilament light chain, improved motor performance, and an extension of life expectancy [38]. Moreover, FP802 protects neurons in human brain organoids derived from ALS patients [38]. FP802 acts via a new pharmacological mode of action, disrupting a death signaling complex consisting of the NMDA receptor (NMDAR) and TRPM4 [36, 37]. Thus, it detoxifies the extrasynaptic NMDAR, one of the key promoters of glutamate neurotoxicity that has long been known to contribute to ALS disease progression [26, 27, 33]. This study aims to evaluate the efficacy of FP802 in preventing retinal degeneration in the SOD1^G93A^ mouse model of ALS.

## Materials and methods

### Animals

The ethical guidelines established by the municipal government (Regierungspräsidium Karlsruhe, Germany) were strictly adhered to throughout the animal experimentation process. This study was approved by the animal care and use committee, Regierungspräsidium Karlsruhe, Referat 35, Germany, AZ 35.9185.81/G-190/19. Heterozygous SOD1^G93A^ transgenic mice expressing the human SOD1^G93A^ mutant on a C57BL/6 J background (www.jax.org/strain/004435) were utilized. The maintenance of heterozygosity involved breeding heterozygous transgenic males with C57BL/6 wild-type females, a service provided by the Interdisciplinary Neurobehavioral Core (INBC) at Heidelberg University. All experiments employed both transgenic and age-matched (20 weeks) wild-type male mice, which were group-housed (up to three mice per cage) at the INBC, where pattern electroretinogram (pERG) experiments took place. Standard cages (15 cm × 21 cm × 13.5 cm) under a 12:12 h light:dark cycle, with unrestricted access to food, water, and nesting material, were provided for all mice. Treatment assignments were randomized within litter blocks. ALZET pumps (ALZET 2004) were filled with either vehicle (40% propylene glycol, P4347, Sigma-Aldrich) or FP802 (166 mg/ml in 40% propylene glycol) and placed in sterile saline at 37 °C for 48 h to equilibrate. Mice were anesthetized with isoflurane inhalation for the entire surgery, with the depth of anesthesia confirmed by the loss of pedal reflex. The insertion site (back of the mouse) was shaved and cleaned with an antiseptic solution, before making a small incision (about 1 cm) with a disinfected scissor. A subcutaneous pocket was then created using blunt, sterile forceps to a size large enough to accommodate the pump. The osmotic pump was then inserted with the releasing side to the opposite direction of incision before closing the incision. The mouse was then placed in a warm recovery cage to maintain body temperature until it regained consciousness. Mice were subsequently monitored for two days to ensure the incision did not reopen. The experimenter remained blinded to the experimental group assignment from implantation until completion of all analyses. A total of 46 heterozygous SOD1^G93A^ and 16 wild-type littermates were used in this study. For the pERG measurement, 10 wild-type and 26 SOD1^G93A^ mice at 20 weeks were used with one SOD1^G93A^ excluded as it died during the pERG measurement. The left and right eyes were collected on the same day after pERG measurement for immunostaining with Brn3A and qPCR analysis, respectively. Another six wild-type and 11 SOD1^G93A^ mice at 20 weeks were used for the determination of the thickness of the retinal nerve fiber layer (RNFL). In addition, 8 SOD1^G93A^ mice at 15 weeks were sacrificed without any treatment for the quantification of RGCs.

### Measurement of FP802 concentrations in the retina

FP802 measurements using mass spectrometry were established by Charles River Laboratories (CRL) (Groningen, Netherland). In mice that were not given FP802, FP802 was neither detected in serum samples nor in brain tissue. The lower limit of quantification (LLOQ) of the methodology used by CRL was 1 nM in serum and 3 pmol/g for brain tissue. To determine FP802 penetrance of the retina, osmotic pump insertion was carried out in wild-type mice according to the same procedure as described for the SOD1^G93A^ mice at the same dose of 40 mg/kg/day. The concentration of FP802 in the retina was measured after 10 days of continuous supply of FP802 using the osmotic pump. Mice were perfused with 20 ml cold PBS to avoid contamination by FP802 present in the blood. The eyes were removed and washed 3 times in cold PBS before the retina was dissected and washed again 3 times in cold PBS. Retinas were frozen on dry ice before being submitted for analysis of FP802 concentration.

### Retinal whole-mount preparation and histology

Retinas were prepared from animals euthanized by an intraperitoneal dose of 400 mg/kg BW pentobarbital (Narcoren; Merial, Hallbergmoos, Germany). The eyes were removed and fixed in 10% formalin for 15 min. The cornea, iris, lens, and vitreous body were then removed under a stereomicroscope. The retinas were dissected by being separated from the pigment epithelium and sclera. For retinal whole-mount immunolabelling, retinas were post-fixed in 10% formalin for 15 min before being placed in PBS. Retinal whole-mounts were then immunolabelled using the free-floating method. Retinas were blocked at room temperature in 1% Triton X-100, 10% FBS and 0.02% sodium azide in PBS for 6 h, followed by overnight incubation with mouse anti-Brn3a (1:200; sc-8429; Santa Cruz Biotechnology, Dallas, TX, USA) at room temperature. Following washing 3 times in PBS, goat anti-mouse IgG (H + L) Alexa Fluor 594 secondary antibody (1:500; A11005, Life Technologies) was applied and incubated overnight at 4 °C. Retinas were then washed again, cut, and mounted onto slides. For each whole-mount, images were equally obtained from eight fields (375 μm × 375 μm) to minimize the influence of location-associated variability in RGC density on cell counts. Retinal whole-mount images were obtained using Las X software via an HC PL APO 20 × objective on a Leica TCS SP8LIA in a DM6 CFS upright confocal microscope. Brn3a-positive cells were detected and quantified automatically using a self-developed macro in CellProfiler with subsequent manual review to eliminate false cell recognition due to non-specific staining. Data analysis was conducted in a single-blind manner, ensuring unawareness of the group assignments.

### Retinal layer measurements

To prepare retinas for sectioning, eyes were enucleated from euthanized mice and prepared as previous described [21]. Briefly, eyes were placed in modified Davidson’s fixative (10% glacial acetic acid, 20% neutral buffered formalin, 30% ethanol, 40% dH_2_O) at room temperature with gentle shaking for 20 h. Following a further 20 h in 4% paraformaldehyde, eyes were washed in PBS and a window created in the posterior wall using a dissecting microscope (Leica, Wetzlar, Germany). After washing in PBS, eyes were placed in 30% ethanol for 1 h and further dehydrated before embedding in paraffin. 0.5 µm paraffin sections were cut using a microtome (Leica SM2000R) and stained with haematoxylin–eosin (H&E) for morphological visualization of retinal layers. Retinal sections traversing the optic nerve head were used for quantification. Images were taken on an Eclipse 80i upright microscope fitted with a DXM1200C camera (Nikon GmbH, Düsseldorf, Germany) using a 10 × objective. RNFL thickness was assessed using ImageJ (NIH) and defined as extending from the inner limiting membrane of the retina to the outer border of the ganglion cell layer. Measurements were taken at 200 µm intervals from the center of the optic disc in both directions and averaged for each eye.

### Gene expression analysis

Following excision of retinas as described above, they were immediately shock-frozen in liquid nitrogen. RNA was isolated using the RNeasy Plus Mini Kit (Qiagen; catalog no.: 74106) with additional on-column DNase I digestion according to the manufacturer's instructions. For complementary DNA synthesis, RNA was reverse transcribed with the High-Capacity cDNA Reverse Transcription Kit (Applied Biosystems; catalog no.: 4368813). Quantitative real-time reverse-transcription (qRT–PCR) was performed on a StepOnePlus thermal cycler using TaqMan gene expression assays (Applied Biosystems; catalog no.: 4331182) for the following genes: *Bdnf* (exon IV; Mm00432069_m1), *cFos* (Mm00487425_m1), *Npas4* (Mm00463644_m1), and *Inhibin beta A* (Mm00434338_m1). Expression levels of target genes evaluated using TaqMan reagents were normalized to the expression of the housekeeping gene, glucuronidase, beta (*GusB*) (Mm00446953_m1), and to the expression levels in WT.

### Electroretinogram recordings

pERG recordings were performed using a UTAS Visual Diagnostic System (LKC Technologies, Gaithersburg, MD, USA) as previously described [28]. Mice were dark-adapted for 30 min prior to recording and were subsequently anesthetized with an intraperitoneal injection of ketamine (100 mg/kg; Atarost GmbH and Co., Twistringen, Germany) and xylazine (10 mg/kg; Albrecht, Aulendorf, Germany). Anesthetized mice were placed on a heating pad to maintain body temperature at 37 °C and were centered at 15 cm in front of a 17-inch cathode ray tube. In order to achieve maximum focus of the pattern stimulation, pupils were not dilated. A transparent contact lens electrode with gold contact (LKC Technologies) was placed on the corneal surface, and both electrical contact and prevention of eye desiccation was facilitated through application of saline solution. Reference and ground needle electrodes were placed subcutaneously in the neck and tail, respectively. Vertical square wave grating stimulations (90% contrast, temporal frequency of 1 Hz equivalent to two pattern reversals per second) were presented at different spatial frequencies (0.05, 0.1, 0.2 and 0.4 cyc/deg), and noise levels were measured with the monitor turned off. Due to the small pERG amplitudes, 100 sweeps were averaged to increase the signal-to-noise ratio, band-pass filtered between 0.3 and 300 Hz, and amplified (64 × gain). pERG amplitude (μV) was measured as the amplitude of the second harmonic of the fast Fourier transformation plotted against spatial frequency of stimulation.

### Statistics

GraphPad Prism 7 software (GraphPad Software) was used to calculate, plot, and evaluate mean values and standard deviation (SD). Data was evaluated for normalcy using a Shapiro–Wilk Test, followed by the statistical tests as outlined in the figure legend. Mean values are shown as bars, and the error bars represent the SD. The following values denote statistical significance: ∗*p* < 0.05, ∗∗*p* < 0.01.

## Results

**Fig. 1:**
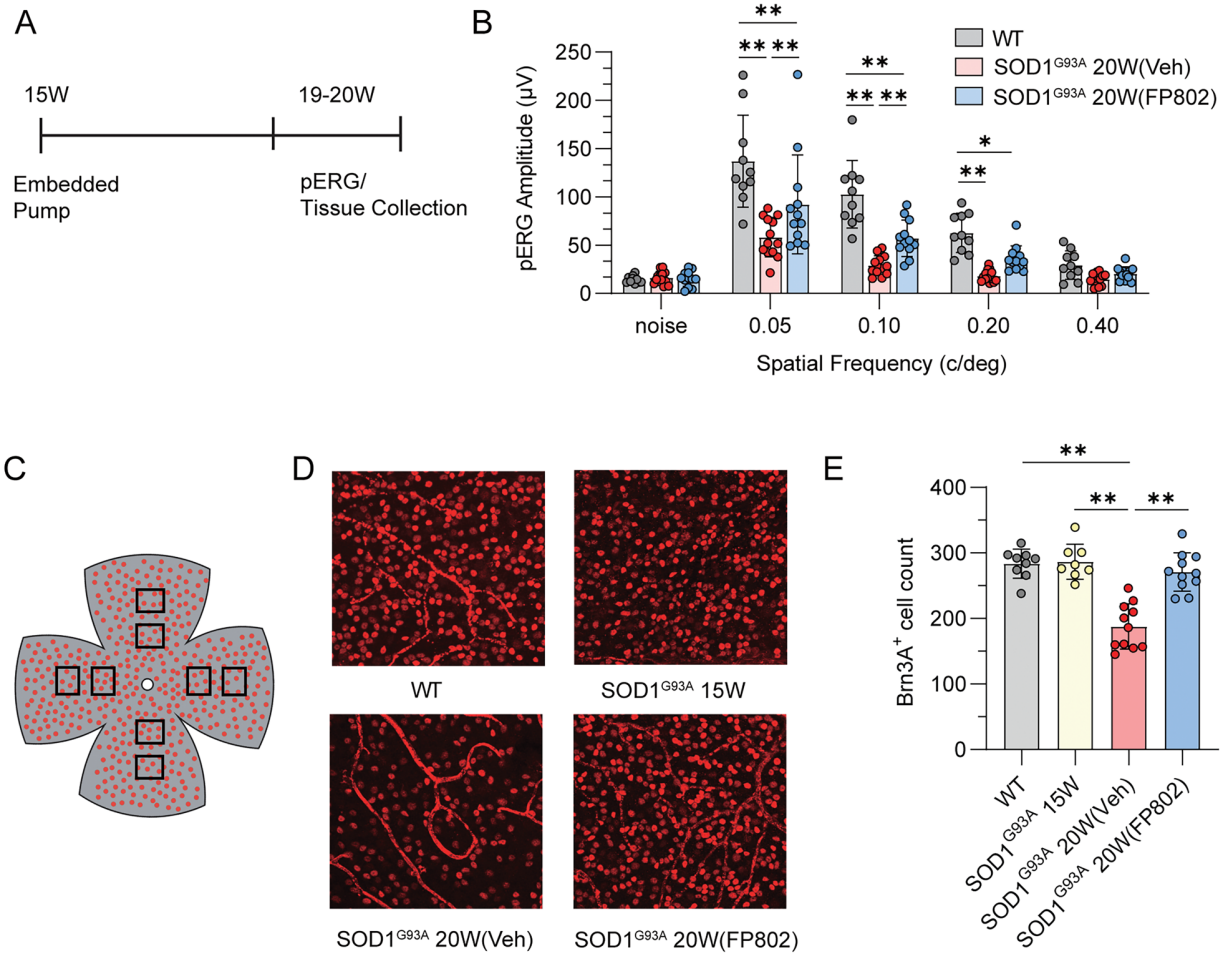
TwinF interface inhibitor FP802 protects against RGC degeneration in the SOD1^G93A^ ALS mouse model. **A** Schematic of experimental protocol followed, with pump implantation (vehicle or FP802) into SOD1^G93A^ transgenic mice from week 15, and subsequent pERG recordings and tissue collection on the same day at weeks 19–20. **B** Quantification of pattern-evoked ERG amplitudes (µV) measured as the amplitude of the second harmonic of the fast Fourier transformation plotted against spatial frequency of stimulation in wild-type (WT), SOD1^G93A^ vehicle-treated and SOD1^G93A^ FP802-treated mice at week 20 (20W). Statistical test: two-way analysis of variance (ANOVA) with Uncorrected Fisher’s LSD for multiple comparisons. n, WT = 10; SOD1^G39A^ 20W (Veh) = 13; SOD1^G93A^ 20W (FP802) = 12. Individual data points represent single eyes per mouse. ∗*p* < 0.05, ∗∗*p* < 0.01. **C** Schematic of retinal whole-mount with squares showing the positioning of areas quantified across each quadrant. **D** Representative images of Brn3a immunolabelling of retinal whole-mounts from mouse groups at 15 and 20 weeks and following treatment with vehicle or FP802 as indicated. **E** Quantification of surviving Brn3a-positive RGCs in retinal whole-mounts (averaged across the retina as indicated in **C**), with cell counts given as the number of Brn3a-positive RGCs quantified per field of view (375 μm × 375 μm). Statistical test: one-way analysis of variance (ANOVA) with post-hoc Tukey’s multiple comparisons test. n, WT = 9; SOD1^G93A^ 15W = 8; SOD1^G93A^ 20W (Veh) = 11; SOD1^G93A^ 20W (FP802) = 11. Single eyes per mouse. ∗*p* < 0.05, ∗∗*p* < 0.01

In our previous study, we described the neuroprotective benefits of FP802 on spinal motor neurons of SOD1^G93A^ mice [38]. Our latest results reveal a significant alleviation of neuro-ophthalmologic symptoms in FP802-treated SOD1^G93A^ mice. FP802 was applied at 40 mg/kg/day using an ALZET osmotic mini pump that was implanted subcutaneously on week 15 and remained until the animals were sacrificed (Fig. 1A). In mice that did not receive FP802, the levels of FP802 in serum and brain tissue using mass spectrometry were undetectable; LLOQ for serum and brain tissue are 1nM and 3 pmol/g, respectively (see also Materials and Methods). However, in those receiving FP802 by ALZET pump delivery, it was confirmed that FP802 effectively crossed the blood-retinal barrier, achieving retinal concentrations of 22.0 ± 10.2 nmol/g (n = 3), equivalent to approximately 12–32 µM.

pERG recordings, acquired 4 to 5 weeks after the onset of FP802 treatment, and subsequent tissue collection were done on the same day (Fig. 1A). pERG recordings were made following pattern stimulation in order to assess the retinal function specifically of RGCs [3]. We found that in wild-type mice, the evoked amplitude of the second harmonic of the stimulus was highest at the lowest pattern spatial frequency and decreased with increasing spatial frequency (Fig. 1B). In SOD1^G93A^ mice receiving only vehicle, the amplitudes for all spatial frequencies (except 0.4 c/deg) were significantly reduced compared to wild-type mice (Fig. 1B). FP802 treatment partially rescued the functional visual deficits in SOD1^G93A^ mice (Fig. 1B).

We next investigated the possibility that neuroprotection of RGCs is responsible for therapeutic benefits of FP802 on retinal function in SOD1^G93A^ mice. We found that compared to 15 weeks old SOD1^G93A^ mice and wild-type mice, the number of RGCs is dramatically lower in 20-weeks-old control vehicle-treated SOD1^G93A^ mice (Fig. 1C–E). This decrease in RGC numbers was virtually completely blocked in 20-weeks-old SOD1^G93A^ mice treated for 4 to 5 weeks with FP802, indicating that similar to the protection of spinal motor neurons [38]. FP802, demonstrated to target the NMDAR/TRPM4 death signaling complex [37], also prevented the loss of RGCs of SOD1^G93A^ mice (Fig. 1D, E).

**Fig. 2:**
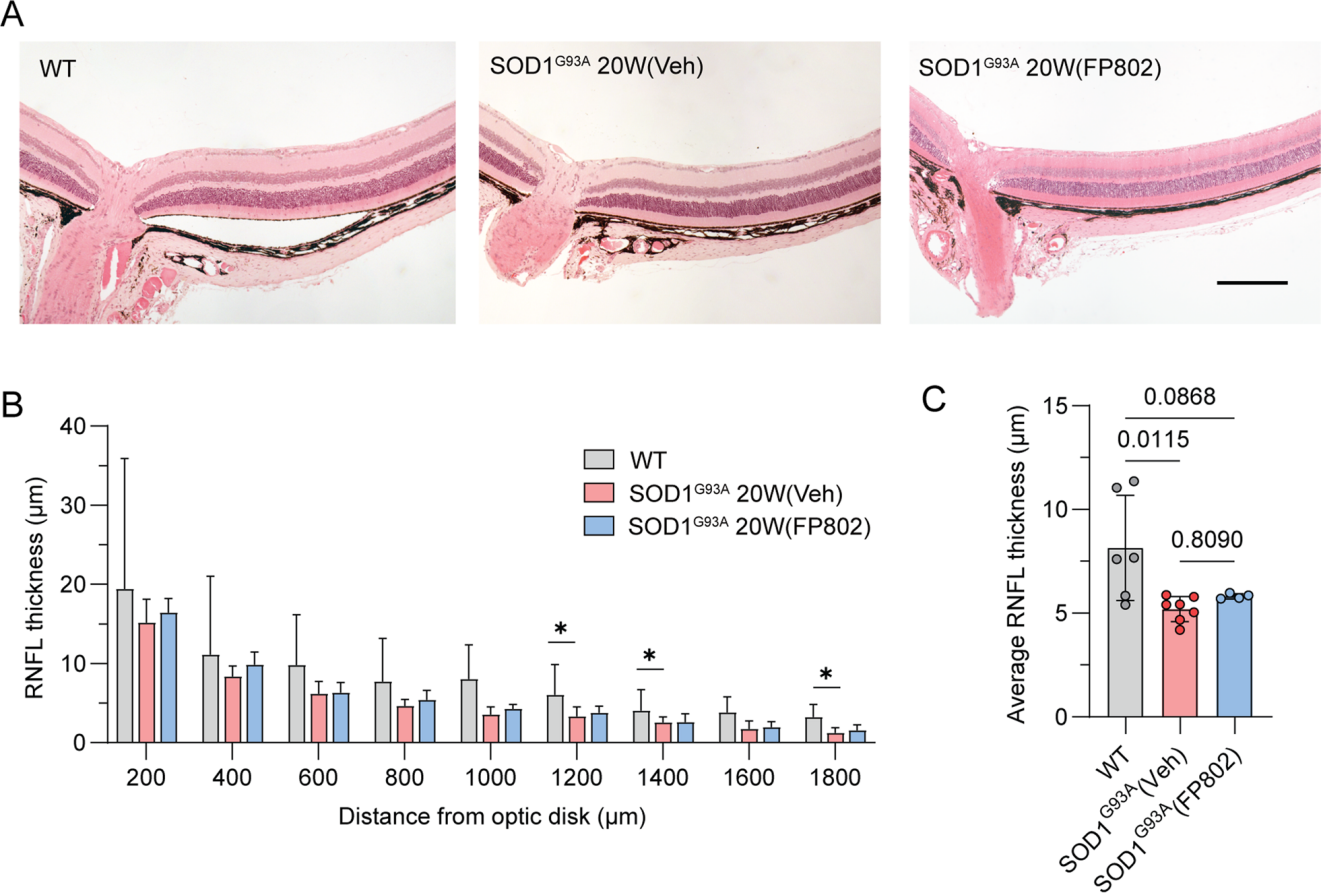
Assessment of RNFL thickness in the untreated and FP802-treated SOD1^G93A^ ALS mouse model. **A** Representative images of 0.5 µm thick paraffin-embedded retinal sections stained with H&E from WT, SOD1^G93A^ 20W (Veh) and SOD1^G93A^ 20W (FP802) mice (scale bar = 200 µm). **B** RNFL thickness was measured with increasing distance from the center of the optic disk. Statistical test: two-way ANOVA with post-hoc Tukey’s multiple comparisons test. **C** The average thickness of the RNFL over the whole distance of 1800 µm from the center of the optic disk was calculated using measurements taken at 200 µm intervals. Statistical test: two-way ANOVA with post-hoc Tukey’s multiple comparisons test. n (**B, C),** WT = 6; SOD1^G93A^ 20W (Veh) = 7; SOD1^G93A^ 20W (FP802) = 4. Single eyes per mouse. ∗*p* < 0.05

To complement the assessment of RGC cell death in SOD1^G93A^ mice, we next evaluated the thickness of the RNFL. This inner retinal layer is comprised of the unmyelinated RGC axons, which converge at the optic disk to form the optic nerve. Eyes from SOD1^G93A^ mice, age-matched wild-type controls, as well as SOD1^G93A^ mice receiving FP802, were excised and sections traversing the optic disc were stained with haematoxylin and eosin (Fig. 2A). The RNFL is thickest near the optic disk, decreasing in size towards the *ora serrata* (Fig. 2B). The RNFL of the vehicle-treated mice was significantly reduced compared to wild-type mice at the following distances from the optic nerve head: 1200 µm, 1600 µm and 1800 µm (Fig. 2B), whereas FP802-treated mice were not significantly different from wild-type controls at any location. This was reflected in the average RNFL thickness, where there was also a significant reduction only in the vehicle-treated SOD1^G93A^ mice compared to wild-type control (Fig. 2C).

**Fig. 3:**
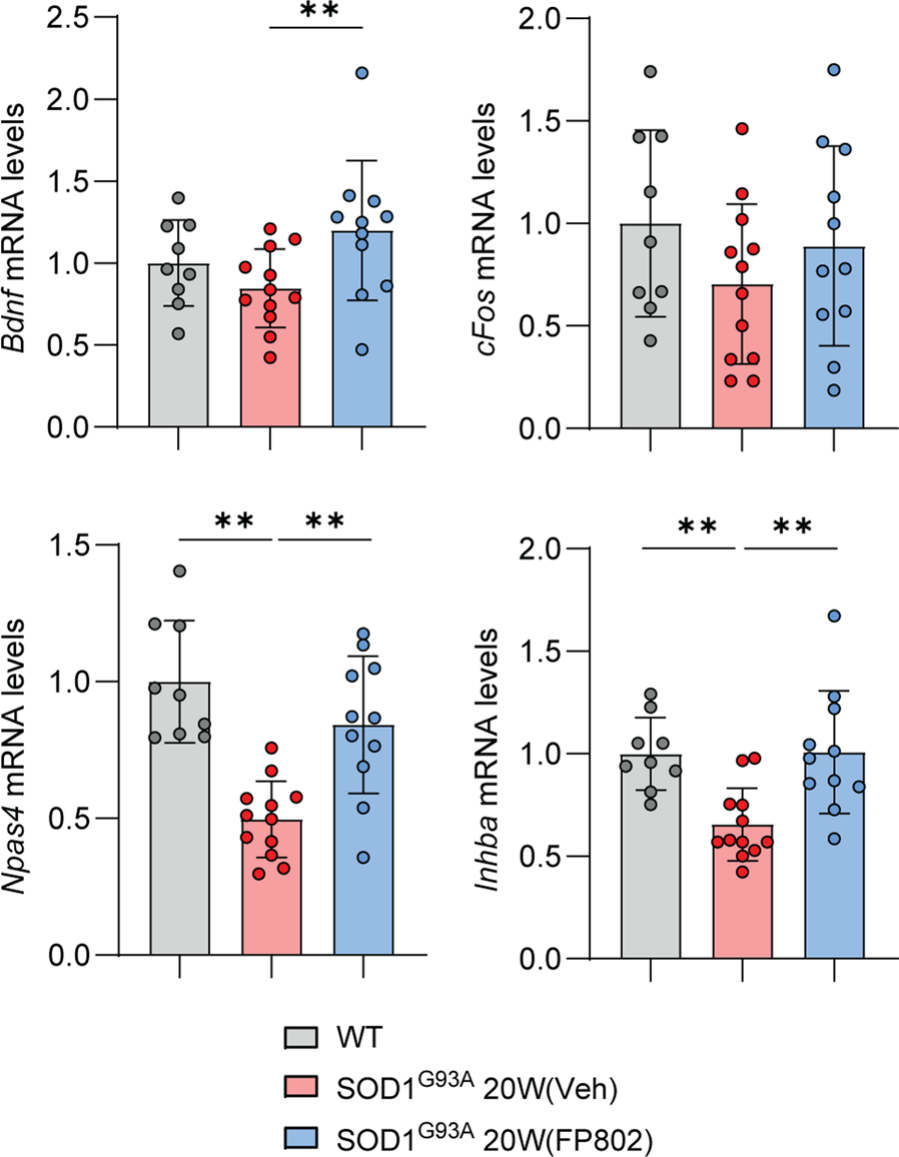
FP802 restores deregulated retinal gene expression in the SOD1^G93A^ ALS mouse model. mRNA levels of *Bdnf*, *cFos*, *Npas4* and *Inhibin beta A* were assessed by RT q-PCR in WT, SOD1^G93A^ vehicle-treated, and SOD1^G93A^ FP802-treated mice at 20 weeks. Statistical test: one-way analysis of variance (ANOVA) with post-hoc Tukey’s multiple comparisons test. n, WT = 9; SOD1^G93A^ 20W (Veh) = 12; SOD1^G93A^ 20W (FP802) = 11. Single eyes per mouse. ∗*p* < 0.05, ∗∗*p* < 0.01

We finally investigated retinal gene expression (Fig. 3). The deregulation of gene transcription is a key component of the pathological triad, which further includes structural disintegration and mitochondrial dysfunction and represents the hallmarks of toxic extrasynaptic NMDAR signaling [2]*.* Specifically, the CREB shut-off pathway induced by extrasynaptic NMDARs [14] in conjunction with the concomitant translocation of class IIa histone deacetylases (HDACs) to the nucleus [8] and inactivation of the ERK/MAP kinase pathway [15] can lead to transcriptional repression of many genes [2, 13]. This repression includes crucial factors such as *Bdnf* and several immediate early genes [14, 37]. qRT-PCR using total retinal lysates revealed that mRNA expression levels of the immediate early genes *Inhibin beta A* and *Npas4* were significantly lower in 20-week-old vehicle-treated SOD1^G93A^ mice compared to wild-type controls (Fig. 3). Moreover, though not significant, the CREB shut-off gene *Bdnf* was reduced considerably (Fig. 3). Treatment of SOD1^G93A^ mice with FP802 increased the expression of *Bdnf* whilst restoring the expression of *Inhibin beta A* and *Npas4* (Fig. 3). The method used to assess gene expression did not allow us to determine which cell types in the retina are subject to transcriptional downregulation. However, it is likely that RGCs are among the affected cell types, as they are vulnerable in SOD1^G93A^ mice and undergo extrasynaptic NMDAR-dependent cell death, which can be blocked by disrupting the NMDAR/TRPM4 complex with FP802 in a manner similar to blocking transcriptional deregulation (see Fig. 1D, E).

## Discussion

Significant progress has recently emerged in ALS treatment, highlighted by the promising results of FP802 that has shown neuroprotective benefits in the SOD1^G93A^ mouse model for ALS. Our previous research has demonstrated the efficacy of FP802 in protecting spinal motor neurons [38]. The results of this study suggest that the benefits of FP802 also extend to the mitigation of neuro-ophthalmologic symptoms. Although ALS patients do not commonly complain of visual impairments (possibly reflecting that the gradual deterioration in vision may not be fully appreciated given the gravity of the other more debilitating symptoms experienced), several studies have reported evidence of decreased visual acuity including impairments in both color and contrast sensitivities [4, 11, 18]. However, visual acuity changes in ALS remain controversial, with other studies not revealing any significant differences between ALS patients and healthy individuals [19, 34]. In contrast, structural changes in the retina have been extensively documented [7, 25, 30, 34], but may in many cases remain below the threshold to significantly affect visual performance. Our findings support the view that FP802 exerts a more widespread protection of neurons than just motor neurons in a model of ALS [38].

There have been some studies into the mechanisms underlying RGC degeneration in ALS patients and mouse models, yet the findings remain controversial. RGC degeneration occurs across all forms of ALS and is not confined to SOD1^G93A^. In SOD1^G93A^ mice, SOD1 aggregation-related vacuoles are predominantly found in the inner nuclear layer (INL) and only rarely in the ganglion cell layer (GCL), indicating that SOD1 aggregation is not a primary cause of RGC loss in SOD1^G93A^ mice [23]. However, SOD1 dysfunction or deletion can result in superoxide anion accumulation in the RGC layer, leading to RGC loss [39]. Thus, even with minimal accumulation of misfolded SOD1 in the GCL, its potential role in RGC degeneration cannot be excluded. Additional possible mechanisms include neuroinflammation, impaired nuclear protein import, endoplasmic reticulum stress, vascular regression, mitochondrial dysfunction, and glutamate excitotoxicity [30]. Although glutamate toxicity has been described to occur in ALS [26, 27, 33], it is not yet clear exactly which type of glutamate receptor is responsible for the toxic effects. Some studies have demonstrated a specific role of the NMDAR in mediating neurodegeneration in animal models of ALS [9, 12, 16, 35], while others describe an involvement of AMPA-type glutamate receptors [10, 20, 31, 33]. Specifically in the ALS retina, the receptors initiating cell toxicity have remained unaddressed. In this study, utilizing a TwinF interface inhibitor, we have demonstrated that NMDAR-mediated glutamate excitotoxicity may be a significant contributor to RGC loss in ALS, since application of FP802 targeting the NMDAR/TRPM4 death complex could halt RGC loss in the SOD1^G93A^ mouse model.

Administered via a subcutaneously implanted ALZET osmotic mini pump starting from week 15, FP802 could prevent the deterioration of visual function in SOD1^G93A^ mice. The mechanism underlying FP802’s impact on retinal function involves the preservation of RGCs, which are crucial for visual processing. The RGC count in FP802-treated SOD1^G93A^ mice remained significantly higher than in their untreated counterparts. Similarly, the pERG responses were also preserved in the FP802-treated SOD1^G93A^ mice, which may directly reflect the increased survival of RGCs since they have been shown to be the origin of this electrophysiological parameter [3]. These findings parallel the protective effects observed in spinal motor neurons [38], suggesting that the NMDAR/TRPM4 death signaling complex is the principal mediator of ALS-associated loss of both spinal motor neurons and RGCs.

Unlike the effect of FP802 on RGC number, the effect of FP802 on the RNFL thickness was not as discernable. Here, although we could detect a significant decrease in the average RNFL thickness (which was most apparent towards the *ora serrata*) in the SOD1^G93A^ mice, there was not a significant difference between the FP802- and vehicle-treated groups. However, the lack of a detectable significant difference following FP802 treatment in comparison to the wild-type controls may suggest a protective effect. This lack of robust effect could reflect the variation inherent in this parameter arising from confounding events such as retinal oedema which may result from on-going neuroinflammation in the tissue [17]. Also, due to regional variations in the RNFL thickness, great care was taken only to measure sections that passed through the optic disk, with precise distance intervals from this location being determined. Future studies could increase the reproducibility by tracking the quadrant locations in relation to their nasal orientation.

Our study also explored the impact of FP802 on the deregulation of gene expression, one of the hallmarks of toxic extrasynaptic NMDAR signaling [2]. Consistent with results of previous studies in cortical and hippocampal neurons [37, 38], FP802 treatment similarly disrupted the transcriptional shut-off pathway in the retina of SOD1^G93A^ mice. The increase in expression of crucial neurotrophic and neuroprotective genes, such as *Bdnf*, *Inhibin beta A*, and *Npas4* [1, 6, 22, 40], contributes to the ability of FP802 to counteract ALS disease progression, underscoring its therapeutic potential in neurodegenerative conditions.

The findings of this study have profound clinical implications for ALS, offering hope in a field with limited treatment options [32]. The ability of FP802 to safeguard both spinal motoneurons and RGCs indicate therapeutic benefits across neurological and ophthalmological symptoms. The preservation of RGCs and (RGC-derived) pERG amplitudes highlight new avenues for addressing often overlooked neuro-ophthalmologic aspects of ALS. In addition, the restoration of gene expression suggests the potential of FP802 to reverse a central disease-promoting process. Further research and clinical trials will be essential to evaluate the efficacy and safety of FP802 in human patients.

## Conclusions

Our study demonstrates a severe loss of RGCs in the SOD1^G93A^ ALS mouse model, resulting in significant impairment in RGC function as revealed by pERG measurements, which can be restored by the application of the TwinF interface inhibitor FP802. FP802, which we previously demonstrated to disrupt the NMDAR/TRPM4 death complex thereby detoxifying extrasynaptic NMDARs [37], effectively protects against RGC degeneration, in addition to preserving motor neurons [38]. This highlights FP802's potential as a novel therapeutic targeting diverse facets of neurodegenerative and retinal diseases, potentially extending beyond ALS.

## Data Availability

Any data will be made available upon request.
